# Socioeconomic disadvantage, fetal environment and child development: linked Scottish administrative records based study

**DOI:** 10.1186/s12939-017-0698-4

**Published:** 2017-11-22

**Authors:** Christopher James Playford, Chris Dibben, Lee Williamson

**Affiliations:** 1 Department of Sociology, University of Exeter, Exeter, UK; 2 Administrative Data Research Centre – Scotland, Edinburgh, UK; 3 School of Geosciences, University of Edinburgh, Edinburgh, UK

**Keywords:** Birth weight, Lifecourse/childhood circumstances, Child health, Health inequalities, Socioeconomic

## Abstract

**Background:**

Cognitive development in childhood is negatively affected by socioeconomic disadvantage. This study examined whether differences in fetal environment might mediate the association between family socioeconomic position and child development.

**Methods:**

Data were linked from the Scottish Longitudinal Study, maternity inpatient records and the Child Health Surveillance Programme – Pre School for 32,238 children. The outcome variables were based on health visitor assessment of gross motor, hearing and language, vision and fine motor, and social development. Socioeconomic position was measured using parental social class and highest qualification attained. Random-effects logistic regression models were estimated to account for multiple reviews and familial clustering. Mediation analysis was conducted using the Karlson-Holm-Breen method.

**Results:**

Hearing and language, vision and fine motor, and social development were associated with lower parental social class and lower parental educational qualifications after adjustment for fetal environment. Fetal environment partially mediated the estimated effect of having parents without educational qualifications for hearing and language (β = 0·15; 95% confidence interval (CI) = 0·07, 0·23), vision and fine motor (β = 0·19; CI = 0·10, 0·28) and social development (β = 0·14; CI = 0·03 to 0·25).

**Conclusions:**

Socioeconomic position predicted hearing and language, vision and fine motor, and social development but not gross motor development. For children of parents without educational qualifications, fetal environment appears to contribute to a part of the socioeconomic gradient in child development abnormalities but post-natal environment appears to still explain the majority of the gradient and for other children most of it.

**Electronic supplementary material:**

The online version of this article (10.1186/s12939-017-0698-4) contains supplementary material, which is available to authorized users.

## Background

The cognitive and emotional development of children growing up in circumstances of socioeconomic disadvantage is more likely to be abnormal compared to children raised in more advantaged families [1]. This association has been noted for a range of different developmental attributes including gross motor skills [2], vision and fine motor skills [3], social development [4] and hearing and language development [5]. Atypical child development outcomes are also associated with low birthweight (LBW) [6] including significant motor impairment [7], conductive hearing loss [8], and a wider spectrum of social and behavioural difficulties [9, 10]. Infants born preterm are more likely to have development problems. A large proportion of extremely preterm children will experience significant development delay, cognitive impairments, learning disabilities, and behavioural and emotional problems [11]. Preterm children are also more likely to have fine motor development problems [12].

The aim of this study was to examine the extent to which differences in fetal environment mediate the association between socioeconomic position and child development. Few studies have simultaneously evaluated the role of other determinants of child development [13]. Previous studies have identified that birthweight is associated with family socioeconomic position with the children of parents in manual occupations being more likely to have low or very low birthweight [14, 15]. Our measure of fetal environment include a range of variables which represent pregnancy and delivery complications (including birthweight and preterm birth), mother’s behaviour and the physical condition of the new born child. Birthweight is associated with family socioeconomic position [14, 15]. We hypothesised that children born to disadvantaged parents would be more likely to have developmental abnormalities but that this would be partially mediated by a poorer fetal environment among more disadvantaged families.

## Methods

### Sample

**Fig. 1:**
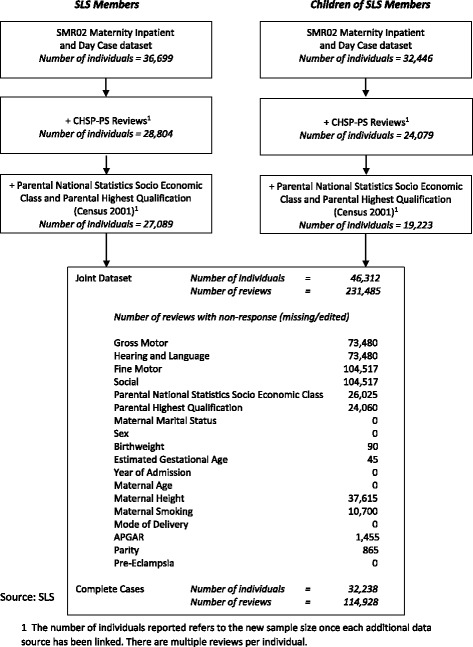
Data Linkage Schema

The data analysed includes a number of administrative datasets linked to the Scottish Longitudinal Study (SLS) by NHS Scotland Information Services Division (ISD). The SLS is a 5.3% representative sample of the Scottish population which links Census records (from 1991, 2001 and 2011) to other administrative data resources [16]. In this study, the linked administrative datasets include the Scottish Morbidity Record maternity inpatient and day case dataset (SMR02) and the Child Health Surveillance Programme – Pre School dataset (CHSP-PS). Details of the linkage are presented in Fig. 1. The mothers of SLS members were identified in the SMR maternity dataset (SMR02) [17]. The sample included all SLS members born from 1991 to 2001 and the children of female SLS members who were born between 1991 and 2005. For both groups, only singleton births were included. Using the relationships between SLS members identified through the Census, it was then possible to include information relating the sex and ethnicity of the individual and their parental occupational classification and parental highest qualification. Not all records were linkable because of the coverage of the CHSP-PS data available,^1^ availability of parental information from the census, and non-response within the datasets. The majority of nonresponse is due to the geographical coverage of the CHSP-PS reviews and differential reviews by area, and therefore was expected. We argue that this is not biasing these results as the non-response is random with regard to the outcomes of interest (this will be discussed further in the limitations).

### Measures

The Child Health Surveillance Programme – Pre-School dataset (CHSP-PS) contains indicators of development which relate to gross motor, hearing and language, vision and fine motor, and social development based on the Woodside system [18]. The classification is based on assessment by a health visitor of the expected progress of children against a series of criteria which are based on the ability of the child to complete a series of tasks [19]. The criteria vary according to the age of the child being assessed. Details of each age-specific assessment are provided in the Child Health Surveillance Programme Pre-School Clinical Guidelines produced by NHS Scotland [19]. Participants are classified as either normal, incomplete, doubtful/uncertain or abnormal.^2^ Doubtful is suggestive of a possible abnormality, rather than an improper response. There are multiple reviews per individual (see Additional file 1: Table S1) and the classification of a child may vary between reviews. The total number of reviews is greater for assessment of Gross Motor and Hearing (114,928) than for Vision and Fine Motor and Social (93,481) because this is recorded in one extra review, the 6–8 week review.

Parental occupation is classified using National Statistics Socio Economic Class (NS-SEC). Analysis has been restricted to natural parents, with legal step-parents excluded. It was decided to use the higher ranking of father’s or mother’s occupation as indicative of familial circumstances. NS-SEC was selected as the measure of socioeconomic position because it is explicitly based the conditions and relations of employment associated with the occupation of an individual [20]. This reflects the material circumstances of the family including the nature of payment contract, prospects for promotion and level of autonomy which are all indicative of socioeconomic position [20]. It is used in all official statistics and surveys in the UK and is a robust and comparable measure.

Parental education is classified using the highest qualification gained, based on the 2001 census. This has been included in these analyses because it captures a different aspect of socioeconomic position, particularly knowledge, skills and the longer term influence of early life circumstances [21]. Sex and ethnicity are recorded in the 2001 census. Ethnicity was not included as an independent variable in the models because the high number of ethnic groups with a low frequency preclude meaningful comparison.

The Maternity Inpatient and Day Case data contains variables recorded at birth including maternal marital status, maternal height, maternal age, maternal smoking status, mode of delivery, parity, APGAR score, birthweight, and estimated gestation. Birthweight percentiles were calculated to control for any changes in the distribution of birthweight over time. Between 1980 and 2003 the proportion of infants born LBW (<2500 g) has remained broadly consistent but the mean birthweight in Scotland has increased [22]. Birthweight centiles were constructed stratified by completed week of estimated gestation and sex based on the sample. When calculating birthweight percentiles, we excluded cases where estimated gestation was less than 32 weeks (i.e. extremely or very preterm) because of a lack of cases. Sensitivity analysis was conducted by estimating the models using birthweight (categorised as <1500 g (Very Low), 1500-2500 g (Low) or >2500 g in line with World Health Organisation guidelines) instead of birthweight percentiles (see Additional file 1: Table S4). Gestational age was divided into three categories (under 32 weeks, 32–37 weeks, 37 weeks or more) to correspond with definitions of very preterm, preterm and early/full-term [23].

### Analyses

The analysis uses a longitudinal design because there were multiple development reviews recorded per child. Random-effects models were fitted because they were more efficient, using all available information from the sample, and also control for unobserved heterogeneity by removing time-invariant error components from the model (i.e. omitted variables).

Two random-effects logistic regression models were estimates for each child development outcome (gross motor, hearing and language, vision and fine motor, and social development) using Stata v13. The first model included parental NS-SEC, parental highest qualification and maternal marital status. The second model included the same variables plus birthweight percentile, estimated gestational age, sex, maternal smoking history, mode of delivery, APGAR score, parity, year of admission, maternal age, maternal height, and pre-eclampsia indicator. Estimates were corrected for familial clustering because the sample included SLS members and the children of SLS members (i.e. siblings were present).

Mediation analysis was conducted using the Karlson-Holm-Breen (KHB) method [24] to decompose the total effect of a socioeconomic measure on the developmental outcome into the direct effect (operating purely via the socioeconomic variable) and the indirect effect (i.e. due to a poorer fetal environment). Unlike linear regression models, direct comparison of the estimated effect sizes is not possible between two logistic regression models because of the fixed variance of the residual. The KHB method works by rescaling the models to ensure comparability. This approach is advantageous because it permits the inclusion of multiple mediators while holding other covariates constant [24].

We therefore specified the KHB models to directly match the approach used in the random-effects modelling. We decomposed the total effect of each socioeconomic variable (parental NS-SEC, parental highest qualification and maternal marital status) on each child development measure (gross motor, hearing and language, vision and fine motor, and social development) into the direct effect and the indirect effect (operating via the fetal environment: birthweight percentile, estimated gestational age, sex, maternal smoking history, mode of delivery, APGAR score, parity, year of admission, maternal age, maternal height, and pre-eclampsia indicator). In the administrative data we have a number of measures of fetal environment: prematurity (estimated gestation), restricted growth (birthweight percentiles), mother’s smoking and a measure of the physical condition of the new born child. These indicators will measure, to some extent, the same causes. This not a problem for this analysis because we are using them together to try and estimate the total impact of fetal exposure to environmental insult and shocks associated with maternal socioeconomic status. We are not trying to attribute this to one particular pathway. We specifically included the group of variables together because we wished to estimate the indirect effect of all of these outcomes simultaneously on the socioeconomic variables.

The hypothesised temporal ordering of the variables is relevant to the mediation model. In this study, parental NS-SEC has been recorded after the birth of the child. We argue that social class as a measure is indicative of long-term socioeconomic position and has been designed to represent a more stable and enduring feature of lifetime circumstances than purely the occupation recorded at time of the census [25], and that this is better recorded later in life as occupational maturity is reached. This analysis uses the higher class position of the parents, which we argue is a better approximation of family circumstances. Parental highest qualification is likely to have been achieved prior to the birth of the child and is also indicative of long-term socioeconomic position.

## Results

Table 1 reports the characteristics of the baseline sample in frequency of reviews. 114,928 Reviews of gross motor and hearing and language development were recorded for 32,238 individuals. The number of reviews of vision and fine motor and social development was lower (93,481 reviews) because these were not reviewed at the 6–8 week review (see Additional file 1: Table S1). There are notable differences in parental socioeconomic position between children with abnormal or doubtful development compared to those with normal development across all indicators. Children with abnormal or doubtful development tend to have parents in less advantaged occupational positions (for all four indicators the *p*-value is less than 0.001, based on Cuzick’s nonparametric test for trend in ordinal data^3^). A similar pattern exists by parental highest qualification.

**Table 1:** Descriptive statistics of the association between child development assessments, socioeconomic position and fetal environment

		Gross Motor		Hearing and Language			Vision and Fine Motor		Social	
	Total sample col. % (n)	Normal	Abnormal/Doubtful	Normal	Abnormal/Doubtful	Total sample % (n)	Normal	Abnormal/Doubtful	Normal	Abnormal/Doubtful
Parental National Statistics Socio Economic Class										
1.1 Large employers and higher managerial	6·3 (7271)	6·3 (7192)	5·3 (79)	6·4 (7072)	4·2 (199)	6·4 (5957)	6·4 (5903)	3·1 (54)	6·4 (5913)	3·2 (44)
1.2 Higher Professionals	7·9 (9094)	7·9 (8991)	6·9 (103)	8 (8846)	5·3 (248)	7·9 (7356)	7·9 (7266)	5·2 (90)	7·9 (7278)	5·7 (78)
2 Lower managerial and professional	28·9 (33176)	28·9 (32785)	26·2 (391)	29·1 (32054)	23·9 (1122)	28·9 (27055)	29·1 (26668)	22·2 (387)	29 (26734)	23·4 (321)
3 Intermediate	15·3 (17597)	15·3 (17399)	13·2 (198)	15·4 (16963)	13·5 (634)	15·3 (14323)	15·4 (14108)	12·3 (215)	15·4 (14169)	11·2 (154)
4 Small employers and own account	5·4 (6233)	5·4 (6166)	4·5 (67)	5·4 (5950)	6 (283)	5·5 (5178)	5·5 (5087)	5·2 (91)	5·5 (5091)	6·3 (87)
5 Lower supervisory and technical	7·9 (9048)	7·9 (8942)	7·1 (106)	7·8 (8630)	8·9 (418)	7·9 (7372)	7·9 (7242)	7·4 (130)	7·9 (7266)	7·7 (106)
6 Semi-routine	14·7 (16892)	14·7 (16639)	16·9 (253)	14·5 (16001)	19 (891)	14·7 (13709)	14·5 (13347)	20·7 (362)	14·6 (13437)	19·8 (272)
7 Routine	8·4 (9701)	8·4 (9522)	12 (179)	8·3 (9181)	11·1 (520)	8·4 (7861)	8·3 (7625)	13·5 (236)	8·3 (7689)	12·5 (172)
Never worked and LTU	3·8 (4311)	3·7 (4219)	6·2 (92)	3·6 (4009)	6·4 (302)	3·7 (3454)	3·6 (3298)	8·9 (156)	3·6 (3335)	8·7 (119)
Full-time students	1·4 (1605)	1·4 (1578)	1·8 (27)	1·4 (1535)	1·5 (70)	1·3 (1216)	1·3 (1192)	1·4 (24)	1·3 (1198)	1·3 (18)
Parental Highest Qualification										
Degree	28·4 (32637)	28·5 (32285)	23·5 (352)	28·7 (31663)	20·8 (974)	28·4 (26506)	28·6 (26196)	17·8 (310)	28·5 (26219)	20·9 (287)
Higher National Certificate/Diploma	11·9 (13623)	11·9 (13464)	10·6 (159)	11·9 (13163)	9·8 (460)	11·8 (11004)	11·8 (10842)	9·3 (162)	11·8 (10872)	9·6 (132)
Highers/Certificate of Sixth Year Studies	18·1 (20761)	18·1 (20534)	15·2 (227)	18·1 (19996)	16·3 (765)	18·1 (16904)	18·1 (16642)	15 (262)	18·1 (16714)	13·9 (190)
Ordinary Grade/Standard Grade	29·6 (34032)	29·6 (33531)	33·5 (501)	29·4 (32420)	34·4 (1612)	29·6 (27679)	29·5 (27076)	34·6 (603)	29·6 (27242)	31·9 (437)
No Qualifications	12·1 (13875)	12 (13619)	17·1 (256)	11·8 (12999)	18·7 (876)	12·2 (11388)	12 (10980)	23·4 (408)	12 (11063)	23·7 (325)
Marital Status										
Married	62·2 (71455)	62·2 (70603)	57 (852)	62·3 (68716)	58·4 (2739)	63·3 (59176)	63·5 (58250)	53·1 (926)	63·4 (58409)	55·9 (767)
Other	37·8 (43473)	37·8 (42830)	43 (643)	37·7 (41525)	41·6 (1948)	36·7 (34305)	36·5 (33486)	46·9 (819)	36·6 (33701)	44·1 (604)
Sex of Child										
Male	51·3 (58984)	51·2 (58114)	58·2 (870)	50·5 (55724)	69·6 (3260)	51·3 (47996)	51 (46827)	67 (1169)	51 (47022)	71 (974)
Female	48·7 (55944)	48·8 (55319)	41·8 (625)	49·5 (54517)	30·4 (1427)	48·7 (45485)	49 (44909)	33 (576)	49 (45088)	29 (397)
Birthweight										
Greater than 2500 g	95·4 (109666)	95·5 (108381)	86 (1285)	95·6 (105342)	92·3 (4324)	95·4 (89178)	95·5 (87638)	88·3 (1540)	95·5 (87954)	89·3 (1224)
Low (1500 g–2500 g)	4·1 (4757)	4·1 (4595)	10·8 (162)	4 (4441)	6·7 (316)	4·1 (3878)	4 (3711)	9·6 (167)	4·1 (3758)	8·8 (120)
Very low (<1500 g)	0·4 (505)	0·4 (457)	3·2 (48)	0·4 (458)	1 (47)	0·5 (425)	0·4 (387)	2·2 (38)	0·4 (398)	2 (27)
Birthweight Percentile^a^										
0–3	2·9 (3347)	2·9 (3253)	6·3 (94)	2·8 (3139)	4·4 (208)	2·9 (2733)	2·9 (2631)	5·8 (102)	2·9 (2639)	6·9 (94)
4–10	6·8 (7833)	6·8 (7692)	9·4 (141)	6·8 (7461)	7·9 (372)	6·8 (6371)	6·8 (6201)	9·7 (170)	6·8 (6252)	8·7 (119)
11–20	9·9 (11324)	9·8 (11161)	10·9 (163)	9·8 (10851)	10·1 (473)	9·8 (9207)	9·8 (9016)	10·9 (191)	9·8 (9040)	12·2 (167)
21–80	59·9 (68788)	59·9 (67993)	53·2 (795)	60 (66161)	56 (2627)	59·9 (56011)	60 (55073)	53·8 (938)	60 (55268)	54·2 (743)
81–90	10 (11547)	10·1 (11426)	8·1 (121)	10 (11070)	10·2 (477)	10·1 (9395)	10·1 (9233)	9·3 (162)	10·1 (9270)	9·1 (125)
91–97	7·1 (8163)	7·1 (8076)	5·8 (87)	7·1 (7812)	7·5 (351)	7·1 (6597)	7·1 (6495)	5·8 (102)	7·1 (6526)	5·2 (71)
98–100	2·9 (3351)	2·9 (3309)	2·8 (42)	2·9 (3227)	2·6 (124)	2·9 (2688)	2·9 (2651)	2·1 (37)	2·9 (2664)	1·8 (24)
Estimated Gestation										
Full Term (>36 weeks)	95·3 (109523)	95·4 (108209)	87·9 (1314)	95·4 (105194)	92·4 (4329)	95·3 (89067)	95·4 (87498)	89·9 (1569)	95·3 (87822)	90·8 (1245)
Preterm (32–36 weeks)	4·2 (4830)	4·1 (4701)	8·6 (129)	4·1 (4527)	6·5 (303)	4·2 (3935)	4·1 (3802)	7·6 (133)	4·2 (3837)	7·1 (98)
Very preterm (<32 weeks)	0·5 (575)	0·5 (523)	3·5 (52)	0·5 (520)	1·2 (55)	0·5 (479)	0·5 (436)	2·5 (43)	0·5 (451)	2 (28)
Maternal Age										
<18	1·9 (2199)	1·9 (2179)	1·3 (20)	1·9 (2098)	2·2 (101)	1·9 (1785)	1·9 (1744)	2·3 (41)	1·9 (1754)	2·3 (31)
18–20	4·3 (4969)	4·3 (4917)	3·5 (52)	4·3 (4743)	4·8 (226)	4·3 (4038)	4·3 (3950)	5 (88)	4·3 (3976)	4·5 (62)
20–24	16·8 (19309)	16·7 (18988)	21·5 (321)	16·6 (18345)	20·6 (964)	16·8 (15743)	16·7 (15343)	22·9 (400)	16·8 (15442)	22 (301)
25–29	31·8 (36521)	31·8 (36112)	27·4 (409)	31·8 (35068)	31 (1453)	32·1 (30023)	32·2 (29498)	30·1 (525)	32·2 (29630)	28·7 (393)
30–34	31·3 (35980)	31·3 (35515)	31·1 (465)	31·5 (34678)	27·8 (1302)	31·2 (29187)	31·3 (28721)	26·7 (466)	31·3 (28805)	27·9 (382)
35–39	12·1 (13937)	12·1 (13744)	12·9 (193)	12·1 (13384)	11·8 (553)	11·9 (11107)	11·9 (10910)	11·3 (197)	11·9 (10936)	12·5 (171)
>39	1·8 (2013)	1·7 (1978)	2·3 (35)	1·7 (1925)	1·9 (88)	1·7 (1598)	1·7 (1570)	1·6 (28)	1·7 (1567)	2·3 (31)
Maternal Smoking										
Never	62 (71281)	62 (70374)	60·7 (907)	62·3 (68629)	56·6 (2652)	61·9 (57899)	62·1 (56999)	51·6 (900)	62·1 (57171)	53·1 (728)
Current	25·9 (29748)	25·9 (29328)	28·1 (420)	25·6 (28246)	32 (1502)	26 (24260)	25·8 (23632)	36 (628)	25·8 (23774)	35·4 (486)
Former	9·4 (10760)	9·4 (10639)	8·1 (121)	9·4 (10380)	8·1 (380)	9·4 (8742)	9·4 (8586)	8·9 (156)	9·4 (8629)	8·2 (113)
Not known	2·7 (3139)	2·7 (3092)	3·1 (47)	2·7 (2986)	3·3 (153)	2·8 (2580)	2·7 (2519)	3·5 (61)	2·8 (2536)	3·2 (44)
Model of Delivery										
Normal	65·2 (74959)	65·3 (74048)	60·9 (911)	65·1 (71807)	67·2 (3152)	65·5 (61228)	65·5 (60089)	65·3 (1139)	65·5 (60320)	66·2 (908)
Other^b^	34·8 (39969)	34·7 (39385)	39·1 (584)	34·9 (38434)	32·8 (1535)	34·5 (32253)	34·5 (31647)	34·7 (606)	34·5 (31790)	33·8 (463)
Parity										
multiparous	54·7 (62861)	54·6 (61951)	60·9 (910)	54·3 (59838)	64·5 (3023)	54·5 (50958)	54·4 (49917)	59·7 (1041)	54·4 (50152)	58·8 (806)
nulliparous	45·3 (52067)	45·4 (51482)	39·1 (585)	45·7 (50403)	35·5 (1664)	45·5 (42523)	45·6 (41819)	40·3 (704)	45·6 (42018)	36·8 (505)
Pre-Eclampsia										
No	91·6 (105279)	91·6 (103933)	90 (1346)	91·6 (100983)	91·7 (4296)	91·4 (85480)	91·5 (83895)	90·8 (1585)	91·5 (84243)	90·2 (1237)
Yes	8·4 (9649)	8·4 (9500)	10 (149)	8·4 (9258)	8·3 (391)	8·6 (8001)	8·5 (7841)	9·2 (160)	8·5 (7867)	9·8 (134)
Observations	100 (114928)	100 (113433)	100 (1495)	100 (110241)	100 (4687)	100 (93481)	100 (91736)	100 (1745)	100 (92110)	100 (1371)
Number of Individuals	32,238					31,731				

Source: SLS

^a^Birthweight percentiles were calculated for all infants born with an estimated gestational age of 32 weeks or more (i.e. these exclude the very preterm)

^b^‘Other’ Mode of Delivery includes all modes of delivery that were not ‘normal, spontaneous vertex vaginal delivery, occipito-anterior.’ For details, see: http://www.ndc.scot.nhs.uk/Dictionary-A-Z/Definitions/index.asp?Search=M&ID=322&Title=Mode%20of%20Delivery%20-%20Babies%201%20to%203

Descriptive statistics for maternal height and year of admission have not been reported

**Fig. 2:**
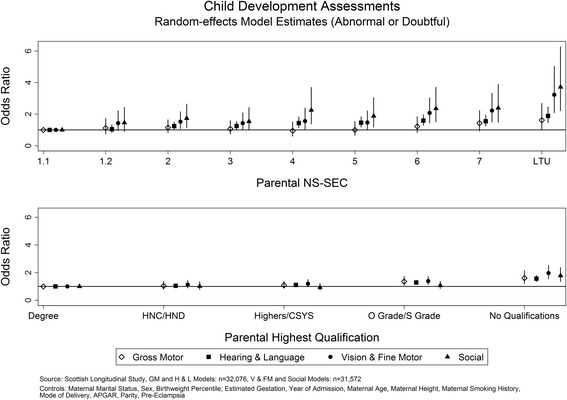
Random effects logistic regression models of development measures and socioeconomic position

Table 2 reports models predicting gross motor, hearing and language, vision and fine motor, and social development. Two models are estimated for each outcome which include measures of social background plus fetal environment.^4^ The patterning of socioeconomic disadvantage varies by child development measure (see Fig. 2). Parental NS-SEC was not associated with gross motor but was predictive of hearing and language, vision and fine motor, and social development. Before adjustment for fetal environment, children whose parents were in semi-routine (NS-SEC 6; OR = 2.18; CI = 1.49, 3.19) and routine occupations (NS-SEC 7; OR = 2.34; CI = 1.56, 3.52) were more likely to have abnormal or doubtful vision and fine motor development than those with parents in higher managerial or professional occupations (NS-SEC 1.1).

**Table 2:** Random effects logistic regression models of development measures with socioeconomic position and fetal environment

	Gross Motor Skills (Abnormal or Doubtful)		Hearing and Language (Abnormal or Doubtful)		Vision & Fine Motor Skills (Abnormal or Doubtful)		Social (Abnormal or Doubtful)	
	Model 1	Model 2	Model 1	Model 2	Model 1	Model 2	Model 1	Model 2
	OR [CI]	OR [CI]	OR [CI]	OR [CI]	OR [CI]	OR [CI]	OR [CI]	OR [CI]
Parent NS-SEC								
1.1	1.00	1.00	1.00	1.00	1.00	1.00	1.00	1.00
1.2	1.17 [0.76,1.82]	1.12 [0.73,1.74]	1.03 [0.81,1.32]	1.05 [0.82,1.34]	1.38 [0.89,2.13]	1.43 [0.93,2.22]	1.43 [0.86,2.39]	1.46 [0.88,2.44]
2	1.15 [0.80,1.67]	1.15 [0.80,1.66]	1.23^*^ [1.01,1.50]	1.24^*^ [1.02,1.51]	1.49^*^ [1.04,2.13]	1.52^*^ [1.06,2.17]	1.73^*^ [1.13,2.64]	1.74^*^ [1.14,2.65]
3	1.06 [0.71,1.59]	1.07 [0.72,1.61]	1.22 [0.98,1.52]	1.25^*^ [1.01,1.55]	1.41 [0.96,2.07]	1.43 [0.98,2.10]	1.49 [0.94,2.36]	1.54 [0.97,2.43]
4	0.98 [0.61,1.57]	0.95 [0.59,1.52]	1.53^***^ [1.19,1.96]	1.44^**^ [1.12,1.84]	1.62^*^ [1.05,2.49]	1.56^*^ [1.02,2.40]	2.33^***^ [1.41,3.84]	2.26^**^ [1.37,3.71]
5	1.03 [0.66,1.59]	1.00 [0.64,1.55]	1.55^***^ [1.23,1.95]	1.46^**^ [1.16,1.84]	1.55^*^ [1.03,2.34]	1.47 [0.97,2.22]	1.94^**^ [1.19,3.17]	1.88^*^ [1.16,3.07]
6	1.24 [0.82,1.88]	1.22 [0.81,1.85]	1.70^***^ [1.37,2.11]	1.59^***^ [1.28,1.98]	2.18^***^ [1.49,3.19]	2.08^***^ [1.42,3.05]	2.46^***^ [1.56,3.87]	2.36^***^ [1.50,3.72]
7	1.45 [0.93,2.26]	1.43 [0.91,2.24]	1.67^***^ [1.32,2.12]	1.55^***^ [1.23,1.96]	2.34^***^ [1.56,3.52]	2.23^***^ [1.48,3.34]	2.52^***^ [1.55,4.11]	2.39^***^ [1.47,3.89]
Never worked and LTU	1.70^*^ [1.03,2.80]	1.62 [0.97,2.70]	2.14^***^ [1.63,2.80]	1.89^***^ [1.44,2.48]	3.60^***^ [2.32,5.58]	3.23^***^ [2.07,5.05]	4.12^***^ [2.46,6.90]	3.72^***^ [2.20,6.28]
FT Students	1.59 [0.82,3.08]	1.65[0.85,3.19]	1.51^*^ [1.05,2.16]	1.78^**^ [1.24,2.56]	1.82 [0.99,3.35]	2.10^*^ [1.13,3.89]	1.98 [0.95,4.13]	2.31^*^ [1.10,4.84]
Parental Highest Qualification								
Degree	1.00	1.00	1.00	1.00	1.00	1.00	1.00	1.00
HNC/HND	1.05 [0.79,1.39]	1.04 [0.78,1.39]	1.07 [0.92,1.25]	1.05 [0.90,1.21]	1.18 [0.91,1.53]	1.13 [0.87,1.46]	1.03 [0.77,1.38]	1.01 [0.76,1.35]
Highers/CSYS	1.07 [0.83,1.38]	1.09 [0.85,1.40]	1.13 [0.98,1.29]	1.11 [0.96,1.27]	1.24 [0.98,1.57]	1.20 [0.95,1.51]	0.93 [0.71,1.22]	0.93 [0.71,1.22]
O Grade/S Grade	1.34^*^ [1.05,1.71]	1.36^*^ [1.06,1.74]	1.40^***^ [1.22,1.59]	1.28^***^ [1.12,1.46]	1.55^***^ [1.25,1.94]	1.39^**^ [1.11,1.73]	1.17 [0.91,1.50]	1.07 [0.83,1.38]
No Qualifications	1.62^**^ [1.21,2.17]	1.61^**^ [1.19,2.16]	1.82^***^ [1.55,2.13]	1.56^***^ [1.33,1.82]	2.40^***^ [1.86,3.09]	1.97^***^ [1.52,2.54]	2.08^***^ [1.56,2.78]	1.78^***^ [1.33,2.38]
Birthweight Percentile								
0–3		2.92^***^ [2.05,4.15]		1.70^***^ [1.38,2.09]		2.08^***^ [1.51,2.87]		2.79^***^ [1.94,4.01]
4–10		1.77^***^ [1.37,2.29]		1.22^**^ [1.05,1.41]		1.44^**^ [1.14,1.80]		1.36^*^ [1.03,1.79]
11–20		1.33^*^ [1.03,1.71]		1.07 [0.93,1.22]		1.14 [0.92,1.42]		1.38^**^ [1.08,1.75]
21–80		1.00		1.00		1.00		1.00
81–90		0.83 [0.64,1.08]		1.07 [0.94,1.22]		1.08 [0.86,1.35]		1.09 [0.84,1.40]
91–97		0.86 [0.63,1.16]		1.15 [0.99,1.34]		0.99 [0.75,1.30]		0.82 [0.59,1.12]
98–100		0.93 [0.59,1.47]		1.00 [0.80,1.25]		0.95 [0.63,1.45]		0.69 [0.42,1.15]
Estimated Gestation								
Full Term (>36 weeks)		1.00		1.00		1.00		1.00
Preterm (32–36 weeks)		2.65^***^ [1.97,3.56]		1.75^***^ [1.49,2.06]		2.21^***^ [1.70,2.86]		1.98^***^ [1.48,2.63]

*Exponentiated coefficients; 95% confidence intervals in brackets*

^*^ *p < 0.05,* ^**^ *p < 0.01,* ^***^ *p < 0.001*

Source: SLS

Model 1 also includes maternal marital status

Model 2 also includes controls for maternal marital status, sex, maternal smoking history, mode of delivery, APGAR score, parity, year of admission, maternal age, maternal height, and pre-eclampsia indicator

Coefficients for other variables included within the models are reported in Additional file 1: Table S3

*Parent NS-SEC* Parental National Statistics Socioeconomic Classification, *Parent Never worked and LTU* Parental has never worked and is long-term unemployed, *Parent FT Students* Parent full time students, *Parent HNC/HND* Parent Higher National Certificate or Higher National Diploma, *Parent Highers/CSYS* Parent Highers or Certificate of Sixth Year Studies, *Parent O Grade/S Grade* Parent Ordinary Grade or Standard Grade

Deficiencies in hearing and language and social development were noted for a wider range of parental NS-SEC classes than for vision and fine motor development. Children whose parents were small employers and own account workers (NS-SEC 4; OR = 1.53; CI = 1.19, 1.96), in lower supervisory and technical occupations (NS-SEC 5; OR = 1.55; CI = 1.23, 1.95), semi-routine occupations (NS-SEC 6; OR = 1.70; CI = 1.37, 2.11) and routine occupations (NS-SEC 7; OR = 1.67; CI = 1.32, 2.12) were more likely to have abnormal or doubtful hearing and language development than those with parents in higher managerial or professional occupations, prior to adjustment for fetal environment (NS-SEC 1.1). Social development followed a similar pattern.

For all four development measures, children with parents that had never worked or were long-term unemployed were more likely to exhibit abnormal or doubtful development than those with parents in higher managerial professional occupations in models which were unadjusted for fetal environment. This was true for gross motor (OR = 1.70; CI = 1.03, 2.80), hearing and language (OR = 2.14; CI = 1.63, 2.80), vision and fine motor (OR = 3.60; CI = 2.32, 5.58), and social development (OR = 4.12; CI = 2.46, 6.90). Children with parents without educational qualifications were more likely than those who parents had a degree to exhibit abnormal or doubtful gross motor (OR = 1.62; CI = 1.21, 2.17), hearing and language (OR = 1.82; CI = 1.55, 2.13), vision and fine motor (OR = 2.40; CI = 1.86, 3.09), and social development (OR = 2.08; CI = 1.56, 2.78) in models which did not adjust for fetal environment.

Infants born in the 0-3rd gestational-age specific percentile for birthweight, were more likely to experience abnormal or doubtful gross motor development (OR = 2.92; CI = 2.05, 4.15), hearing and language (OR = 1.70; CI = 1.38, 2.09), vision and fine motor (OR = 2.08; CI = 1.51, 2.87), and social development (OR = 2.79; CI = 1.94). The effect size was smaller for infants born in the 4th–10th percentiles. We did not observe a greater risk for infants whose gestational-age specific birthweight percentile is above average. Similar results are reported when the analysis was performed using absolute measures of birthweight (see Additional file 1: Table S4). There was also clear association between pre-term birth and gross motor development (OR = 2.65; CI = 1.97, 3.56), hearing and language (OR = 1.75, CI = 1.49, CI = 2.06), vision and fine motor (OR = 2.21; CI = 1.70, 2.86), and social development (OR = 1.98; CI = 1.48, 2.63) (Table 2).

The inclusion of fetal environment measures did not attenuate greatly the association between socioeconomic disadvantage and child development outcomes. For example, when adjusting for fetal environment, the risk of abnormal or doubtful development decreased slightly among children whose parents had never worked or were long term unemployed for gross motor skills (Model 1 OR = 1.70; CI = 1.03, 2.80; Model 2 OR = 1.62; CI = 0.97, 2.70), hearing and language (Model 1 OR = 2.14; CI = 1.63, 2.80; Model 2 OR = 1.89; CI = 1.44, 2.48), vision and fine motor skills (Model 1 OR = 3.60; CI = 2.32, 5.58; Model 2 OR = 3.23; CI = 2.07, 5.05), and social development (Model 1 OR = 4.12; CI = 2.46, 6.90; Model 2 OR = 3.72; CI = 2.20, 6.28), and (Table 2). For robustness, we used the Karlson-Holm-Breen (KHB) method to decompose the total effects observed into direct effects (operating purely via the socioeconomic variable) and indirect effects (i.e. via the fetal environment measures, see Table 3). For the majority of parental NS-SEC classes and levels of parental qualifications, the contribution of indirect effects (i.e. via differential fetal environment) were minimal. However for children in households with parents who have never worked or who have no educational qualifications, around a quarter of the effect associated with socioeconomic status appears to be related to the fetal environment measures (based on comparison of indirect effect with total effect, see Table 3).

**Table 3:** The pathway from parental socioeconomic position to abnormal/doubtful child development via birth factors^a^

	Gross Motor Skills	Hearing & Language	Vision & Fine Motor Skills	Social
	β [CI]	β [CI]	β [CI]	β [CI]
Parent NS-SEC				
1.2 Higher Professionals				
Total effect	0·14 [−0·27,0·55]	0·02 [−0·21,0·26]	0·33 [−0·09,0·75]	0·37 [−0·11,0·85]
Direct effect	0·13 [−0·28,0·54]	0·05 [−0·18,0·29]	0·38 [−0·05,0·80]	0·40 [−0·08,0·89]
Indirect effect	0·01 [−0·05,0·07]	−0·03 [−0·10,0·04]	−0·05 [−0·13,0·03]	−0·03 [−0·12,0·06]
2 Lower managerial and professional				
Total effect	0·14 [−0·20,0·48]	0·20 [0·01,0·39]	0·43 [0·08,0·78]	0·57 [0·16,0·97]
Direct effect	0·16 [−0·18,0·50]	0·22 [0·03,0·40]	0·45 [0·10,0·80]	0·59 [0·19,1·00]
Indirect effect	−0·02 [−0·08,0·04]	−0·02 [−0·09,0·05]	−0·02 [−0·10,0·06]	−0·02 [−0·11,0·07]
3 Intermediate				
Total effect	0·05 [−0·32,0·43]	0·20 [−0·01,0·40]	0·37 [−0·01,0·75]	0·44 [−0·01,0·88]
Direct effect	0·09 [−0·29,0·47]	0·22 [0·02,0·43]	0·40 [0·02,0·78]	0·47 [0·03,0·92]
Indirect effect	−0·04 [−0·11,0·03]	−0·03 [−0·10,0·04]	−0·02 [−0·11,0·06]	−0·03 [−0·13,0·06]
4 Small employers and own account				
Total effect	−0·01 [−0·47,0·44]	0·43 [0·19,0·67]	0·53 [0·10,0·96]	0·90 [0·41,1·39]
Direct effect	0·00 [−0·46,0·45]	0·37 [0·13,0·61]	0·50 [0·06,0·93]	0·87 [0·38,1·36]
Indirect effect	−0·01 [−0·08,0·06]	0·06 [−0·01,0·14]	0·04 [−0·05,0·12]	0·03 [−0·07,0·12]
5 Lower supervisory and technical				
Total effect	0·03 [−0·38,0·45]	0·42 [0·20,0·65]	0·46 [0·05,0·87]	0·68 [0·20,1·15]
Direct effect	0·05 [−0·37,0·47]	0·38 [0·16,0·61]	0·43 [0·02,0·84]	0·67 [0·19,1·14]
Indirect effect	−0·02 [−0·10,0·06]	0·04 [−0·04,0·12]	0·03 [−0·06,0·12]	0·01 [−0·09,0·11]
6 Semi-routine				
Total effect	0·20 [−0·18,0·59]	0·52 [0·31,0·73]	0·81 [0·43,1·19]	0·94 [0·49,1·38]
Direct effect	0·25 [−0·14,0·63]	0·47 [0·26,0·68]	0·79 [0·41,1·18]	0·94 [0·49,1·39]
Indirect effect	−0·04 [−0·13,0·04]	0·05 [−0·03,0·13]	0·02 [−0·07,0·11]	0·00 [−0·11,0·10]
7 Routine				
Total effect	0·35 [−0·06,0·77]	0·51 [0·28,0·73]	0·89 [0·49,1·29]	0·95 [0·48,1·42]
Direct effect	0·40 [−0·01,0·82]	0·44 [0·21,0·67]	0·86 [0·46,1·27]	0·94 [0·47,1·42]
Indirect effect	−0·05 [−0·14,0·04]	0·06 [−0·02,0·14]	0·03 [−0·06,0·13]	0·01 [−0·10,0·12]
Never worked and Long Term Unemployed				
Total effect	0·51 [0·04,0·99]	0·78 [0·52,1·03]	1·32 [0·88,1·76]	1·47 [0·96,1·98]
Direct effect	0·57 [0·08,1·05]	0·65 [0·39,0·91]	1·24 [0·80,1·69]	1·41 [0·90,1·93]
Indirect effect	−0·05 [−0·18,0·08]	0·13 [0·03,0·22]	0·08 [−0·05,0·20]	0·06 [−0·09,0·20]
Full Time Student				
Total effect	0·39 [−0·24,1·02]	0·34 [−0·02,0·71]	0·54 [−0·09,1·18]	0·65 [−0·10,1·39]
Direct effect	0·51 [−0·13,1·15]	0·57 [0·21,0·94]	0·75 [0·12,1·39]	0·87 [0·12,1·62]
Indirect effect	−0·12 [−0·25,0·01]	−0·23 [−0·33,−0·13]	−0·21 [−0·34,−0·08]	−0·22 [−0·37,−0·08]
Parent Highest Qualification				
Higher National Certificate/Diploma				
Total effect	0·05 [−0·22,0·32]	0·07 [−0·08,0·21]	0·14 [−0·11,0·40]	0·02 [−0·27,0·30]
Direct effect	0·06 [−0·21,0·33]	0·05 [−0·10,0·20]	0·12 [−0·13,0·38]	0·01 [−0·28,0·30]
Indirect effect	−0·01 [−0·08,0·05]	0·02 [−0·06,0·09]	0·02 [−0·06,0·10]	0·01 [−0·09,0·10]
Highers/Certificate of Sixth Year Studies				
Total effect	0·04 [−0·20,0·29]	0·12 [−0·01,0·25]	0·20 [−0·03,0·43]	−0·09 [−0·36,0·17]
Direct effect	0·07 [−0·18,0·32]	0·10 [−0·03,0·24]	0·17 [−0·06,0·40]	−0·09 [−0·36,0·17]
Indirect effect	-0·03 [−0·09,0·04]	0·02 [−0·06,0·09]	0·03 [−0·05,0·11]	0·00 [−0·09,0·09]
Ordinary Grade/Standard Grade				
Total effect	0·27 [0·04,0·50]	0·33 [0·21,0·46]	0·43 [0·21,0·65]	0·13 [−0·12,0·37]
Direct effect	0·30 [0·06,0·54]	0·25 [0·12,0·38]	0·32 [0·10,0·54]	0·06 [−0·19,0·31]
Indirect effect	-0·03 [−0·11,0·04]	0·08 [0·01,0·16]	0·10 [0·02,0·19]	0·06 [−0·04,0·16]
No Qualifications				
Total effect	0·45 [0·16,0·74]	0·59 [0·44,0·75]	0·86 [0·60,1·11]	0·71 [0·42,1·00]
Direct effect	0·48 [0·19,0·78]	0·44 [0·29,0·60]	0·67 [0·41,0·93]	0·57 [0·27,0·86]
Indirect effect	-0·03 [−0·12,0·06]	0·15 [0·07,0·23]	0·19 [0·10,0·28]	0·14 [0·03,0·25]

Source: SLS

^a^Based on Model 2 in Table 2. These models also control for maternal marital status. The indirect effect includes birthweight percentile, pre-term indicator, sex, maternal smoking history, mode of delivery, APGAR score, parity, year of admission, maternal age, maternal height, and pre-eclampsia indicator

As part of a wider sensitivity analysis, we also investigated whether there were differentials in the association by the sex of the child (see Additional file 1: Tables S8 and S9). The effect size for socioeconomic position variables for males and females were broadly consistent with the main-effects models estimated in Table 2.

## Discussion

For most children there appears to be no notable socioeconomic pathway through in utero development. However, for children in households in the most socioeconomically challenging situations there is evidence of a small but notable effect. Children growing up in circumstances of socioeconomic disadvantage, manifested through parental occupation and parental education, were more likely to experience abnormal or doubtful vision and fine motor, social, and hearing and language development. Deficits in gross motor skills were not associated with parental occupation but were very slightly more likely among children of parents without educational qualifications or whose highest qualification was O Grade/S Grade. The findings of this study are consistent with Dammann et al. [26] who observed an association between socioeconomic background and intelligence and language skills but not visuomotor development. More recently, Hung et al. identified that children born to socioeconomically disadvantaged parents were more likely to exhibit neurological abnormalities and that this could not be explained by differences in perinatal factors [1]. A meta-analysis of studies investigating gross motor skills and socioeconomic background suggested that findings were inconsistent [27].

The longitudinal dataset created in this study contains information on fetal environment, child development measures, and parental socioeconomic position. MM Black, CR Hess and J Berenson-Howard [28] note that gaps in development by socioeconomic position are typically first observed in toddlerhood. The use of information from a number of reviews from 6 to 8 weeks to 48 months increases the likelihood of correctly measuring development and reduces the problems associated with a single point of observation. A further advantage of using the Scottish Longitudinal Study over other cohort or longitudinal survey datasets is that there is very little loss to follow up. There is also an established history of linking Scottish Morbidity Record (SMR) health data to the SLS. Many previous studies on child development have been based on small sample sizes. Using the SLS is much lower cost and use of secondary data minimises intrusion for data subjects.

The findings of this study suggest that children growing up in circumstances of disadvantage are more likely to have poor development outcomes and this is consistent with other studies [29]. The causal pathways for this relationship are not clear though [30]. It is plausible that socioeconomic disparities in brain structure may explain part of this association [30] and this offers a potential avenue for future study. We cannot identify from the information available within this study where the cause of abnormal or doubtful development was due to genetic conditions or lifestyle factors that pre-date birth (such as alcohol use or use of other drugs which may be detrimental during pregnancy). The developmental measures analysed in this study may be indicative of poor or delayed cognitive skills but also of chronic disabling conditions. N Spencer and L Strazdins [31] and CM Blackburn, NJ Spencer and JM Read [32] have identified an association between socioeconomic disadvantage in early childhood and subsequent development of chronic disabling conditions. What this study may also suggest is that the environment in which children grow up is consequential and that family behaviours differ [33]. Differences in access to resources such as nutrition, access to health care, housing cognitively stimulating materials and experiences and parental expectations and styles have been proposed as possible reasons for the gradient in child development by socioeconomic position [4]. Differential exposure to stress and coping responses to stress are another feasible explanation for the socioeconomic gradient in child development outcomes [4].Without further information on factors occurring in the early years of a child’s life, it is challenging to investigate how these factors moderate, mediate and transact to affect the relationship between socioeconomic position and child development [33].

### Limitations

There is nonresponse within the datasets, particularly with respect to developmental reviews. Wood et al. audited the coverage of CHSP-PS reviews and concluded that there is a progressive decline in review coverage from first to the later reviews and that coverage is lower among deprived groups [34]. However, this has not changed over time. The child development assessments are carried out by a public health visitor and may be used to indicate that a child should be referred as requiring additional support needs. There is a possibility that there may be some misestimation of the underlying condition if the health visitor observes a behaviour but judges that additional support needs will be required or if a health visitor assessment is influenced by the parental socioeconomic position or the child’s perinatal history.

Parental occupation and highest qualification are recorded in the 2001 census. These are a proxy for life circumstances and may not perfectly reflect the exact circumstances for young people in their early years. However, the use of the highest occupation or qualification attained for parents is a fairly reliable indicator of general socioeconomic position.

The specification of the mediation model sought to distinguish the indirect effects of a range of measures of fetal environment relative to post-natal effects of the socioeconomic position context. The measures of fetal environment used in this study (including birthweight and gestational age) will not capture all in utero insults and shocks and therefore we may have inadvertently attributed some in utero effects to post-natal environment because maternal behaviour and environment pre and post-natal are likely to be correlated. We may have therefore under estimated the in utero socioeconomic effect. We do not perceive there to be a reverse causation problem as child development occurs after birth. Whilst there may be some omitted variables bias, we have sought to overcome this by the inclusion of many potential confounders and the use of longitudinal data models. Examples of potential confounders might include measures of mother’s mental health, maternal and child nutrition, parenting patterns, or domestic violence among others. The administrative datasets linked as part of this study did not include indicators covering such measures but future studies might investigate in greater detail these factors, if available.

## Conclusions

After adjustment for measures of fetal environment, socioeconomic position was a strong predictor of development of hearing and language, vision and fine motor skills, and social skills but not gross motor skills. For most children this gradient appears to be produced not in utero but either differences in early life environment or possibly a pathway affecting both a mother’s socioeconomic position and her offspring’s development (e.g. genetic/epigenetic). For children of parents with no educational qualification, a poor fetal environment does appear to make a major contribution to the chance of child development abnormalities but the majority of the effect still appears to be through the post-natal environment.

In order to tackle socioeconomic inequalities in child development for most children, a focus on differences in early life environments may be most important. For children in the poorest socioeconomic position interventions should also focus on both early life environment and the health and behaviours of mothers during pregnancy. Future research should examine other factors in early childhood that may influence child development, such as antenatal and postnatal stress [35], to explore whether these mediate the relationship between socioeconomic disadvantage and child development.

## Additional file

Additional file 1: Table S1. Child Health Surveillance Programme- Pre-school review coverage. **Table S2.** Descriptive statistics of the association between socioeconomic position and fetal environment measures. **Table S3.** Tests of independence between measures of socioeconomic position and fetal environment. **Table S4.** Random effects logistic regression models of development measures with fetal environment (including birthweight percentile). **Table S5.** Random effects logistic regression models of development measures with fetal environment (including birthweight). **Table S6.** Random effects logistic regression models of development measures with socioeconomic position and fetal environment (including estimates not reported in Table 2). **Table S7.** Random effects logistic regression models of development measures with socioeconomic position and fetal environment (sensitivity analysis using birthweight). **Table S8.** Random effects logistic regression models of development measures with socioeconomic position and fetal environment for males and females (1). **Table S9.** Random effects logistic regression models of development measures with socioeconomic position and fetal environment for males and females (2). (DOCX 67 kb)

## Abbreviations

CHSP-PS: Child Health Surveillance Programme – Pre School dataset
ISD: NHS Scotland Information Services Division
KHB: Karlson-Holm-Breen method
LBW: Low Birthweight
NS-SEC: National Statistics Socio Economic Class
SLS: Scottish Longitudinal Study
SMR: Scottish Morbidity Record
SMR02: Scottish Morbidity Record maternity inpatient and day case dataset

## Footnotes

1 For details, see: http://www.isdscotland.org/Health-Topics/Child-Health/Child-Health-Programme/Child-Health-Systems-Programme-Pre-School.asp accessed 01/03/17.

2 For further details, see http://www.isdscotland.org/Health-Topics/Child-Health/Child-Health-Programme/Child-Health-Systems-Programme-Pre-hall4.asp accessed 26/09/17.

3 Whilst this test assumes independence between observations, the broad indication of trend merits further investigation using panel data models reported in Tables 3–6 to correctly adjust for the variance structure.

4 For the results of a sensitivity analysis using birthweight categories instead of birthweight percentiles see Additional file 1: Table S7.
