# Supplementary material for: Socioeconomic disadvantage, fetal environment and child development: linked Scottish administrative records based study
Source: Int J Equity Health. 2017 Nov 22;16:203. doi: 10.1186/s12939-017-0698-4 (PMC5700527; doi:10.1186/s12939-017-0698-4)
Supplement: Additional file 1: Table S1. — Child Health Surveillance Programme- Pre-school review coverage. Table S2. Descriptive statistics of the association between socioeconomic position and fetal environment measures. Table S3. Tests of independence between measures of socioeconomic position and fetal environment. Table S4. Random effects logistic regression models of development measures with fetal environment (including birthweight percentile). Table S5. Random effects logistic regression models of development measures with fetal environment (including birthweight). Table S6. Random effects logistic regression models of development measures with socioeconomic position and fetal environment (including estimates not reported in Table 2). Table S7. Random effects logistic regression models of development measures with socioeconomic position and fetal environment (sensitivity analysis using birthweight). Table S8. Random effects logistic regression models of development measures with socioeconomic position and fetal environment for males and females (1). Table S9. Random effects logistic regression models of development measures with socioeconomic position and fetal environment for males and females (2). (DOCX 67 kb) [file 12939_2017_698_MOESM1_ESM.docx]

**Additional file 1**

**Abbreviations used in supplementary tables:**

Parent NS-SEC Parental National Statistics Socio-Economic Classification

Parent Never worked and LTU Parental has never worked and is long-term unemployed

Parent FT Students Parent full time students

Parent HNC/HND Parent Higher National Certificate or Higher National Diploma

Parent Highers/CSYS Parent Highers or Certificate of Sixth Year Studies

Parent O Grade/S Grade Parent Ordinary Grade or Standard Grade

**Table S1:** Child Health Surveillance Programme- Pre-school review coverage

| **Review** | **Gross Motor / Hearing** | **Fine Motor / Social** |
| --- | --- | --- |
| 6-8 Week Review | 21,446 | - |
| 8 to 9 Month Review | 22,147 | 22,146 |
| 21 to 24 Month Review (pre Hall 4) | 24,488 | 24,488 |
| 39 to 42 Month Review (pre Hall 4) | 24,991 | 24,991 |
| 48 Month Review/ Pre-school (pre Hall 4) | 21,856 | 21,856 |
| Total number of reviews | 114,928 | 93,481 |
| Number of individuals (n) | 32,238 | 31,731 |

Source: SLS

**Table S2:** Descriptive statistics of the association between socioeconomic position and fetal environment measures.

|  |  | Birthweight | | | Birthweight Percentile ^a^ | | | | | | Estimated Gestation | | |
| --- | --- | --- | --- | --- | --- | --- | --- | --- | --- | --- | --- | --- | --- |
|  | Total sample (n) | Greater than 2500g | Low  (1500g-2500g) | Very low (<1500g) | 0-10 | 11-20 | 21-80 | 81-90 | 91-97 | 98-100 | Full Term  (>36 weeks) | Preterm  (32-36 weeks) | Very preterm  (<32 weeks) |
| Parental National Statistics Socio Economic Class |  |  |  |  |  |  |  |  |  |  |  |  |  |
| 1.1 Large employers and higher managerial | 7271 | 7008 | 263 | | 447 | 605 | 4301 | 894 | 696 | 289 | 6929 | 342 | |
| 1.2 Higher Professionals | 9094 | 8790 | 304 | | 678 | 630 | 5510 | 1133 | 825 | 294 | 8768 | 326 | |
| 2 Lower managerial and professional | 33176 | 31943 | 1233 | | 2458 | 2754 | 20413 | 3541 | 2679 | 1172 | 31768 | 1408 | |
| 3 Intermediate | 17597 | 16813 | 784 | | 1617 | 1744 | 10741 | 1822 | 1125 | 470 | 16713 | 884 | |
| 4 Small employers and own account | 6233 | 6019 | 214 | | 497 | 609 | 3920 | 528 | 439 | 207 | 5979 | 254 | |
| 5 Lower supervisory and technical | 9048 | 8550 | 498 | | 1005 | 965 | 5270 | 839 | 605 | 292 | 8496 | 552 | |
| 6 Semi-routine | 16892 | 15927 | 965 | | 2196 | 2055 | 9691 | 1532 | 1016 | 329 | 16044 | 848 | |
| 7 Routine | 9701 | 9136 | 565 | | 1392 | 1149 | 5610 | 807 | 499 | 185 | 9236 | 465 | |
| Never worked and LTU | 4311 | 3959 | 352 | | 703 | 626 | 2403 | 295 | 168 | 83 | 4040 | 271 | |
| Full-time students | 1605 | 1521 | 84 | | 187 | 187 | 929 | 156 | 111 | 30 | 1550 | 55 | |
| Parental Highest Qualification |  |  |  |  |  |  |  |  |  |  |  |  |  |
| Degree | 32637 | 31596 | 928 | 113 | 2206 | 2510 | 19973 | 3808 | 2823 | 1194 | 31391 | 1123 | 123 |
| Higher National Certificate/Diploma | 13623 | 13093 | 489 | 41 | 1085 | 1379 | 8300 | 1395 | 984 | 441 | 12949 | 635 | 39 |
| Highers/Certificate of Sixth Year Studies | 20761 | 19869 | 780 | 112 | 1865 | 1928 | 12520 | 2094 | 1627 | 618 | 19857 | 795 | 109 |
| Ordinary Grade/Standard Grade | 34032 | 32243 | 1603 | 186 | 3919 | 3690 | 20115 | 3202 | 2026 | 859 | 32270 | 1541 | 221 |
| No Qualifications | 13875 | 12865 | 957 | 53 | 2105 | 1817 | 7880 | 1048 | 703 | 239 | 13056 | 736 | 83 |
| Observations | 114928 | 109666 | 4757 | 505 | 11180 | 11324 | 68788 | 11547 | 8163 | 3351 | 109523 | 4830 | 575 |
| Number of Individuals | 32238 |  |  |  |  |  |  |  |  |  |  |  |  |

Source: SLS

For the birthweight by parental NS-SEC frequencies and the estimated gestation by NS-SEC frequencies, the reported frequencies for some categories have had to be collapsed due to statistical disclosure control. The measures of association in online-only table 3 are based on the un-collapsed birthweight and estimated gestation categories.

**Table S3:** Tests of independence between measures of socioeconomic position and fetal environment.

| **Socioeconomic Variable** | **Birth Outcome** | **Pearson’s Chi-square** | **Degrees of Freedom** | **p-value** |
| --- | --- | --- | --- | --- |
| Parental National Statistics Socio Economic Class | Birthweight | 434.9 | 18 | <0.001 |
| Parental National Statistics Socio Economic Class | Birthweight Percentile | 2175.3 | 45 | <0.001 |
| Parental National Statistics Socio Economic Class | Estimated Gestation | 143.4 | 18 | <0.001 |
| Parental Highest Qualification | Birthweight | 478.2 | 8 | <0.001 |
| Parental Highest Qualification | Birthweight Percentile | 1876.4 | 20 | <0.001 |
| Parental Highest Qualification | Estimated Gestation | 153.8 | 8 | <0.001 |

Source: SLS

**Table S4:** Random effects logistic regression models of development measures with fetal environment (including birthweight percentile).

|  | **Gross Motor Skills**  **(Abnormal or Doubtful)** | **Hearing and Language**  **(Abnormal or Doubtful)** | **Vision & Fine Motor Skills**  **(Abnormal or Doubtful)** | **Social**  **(Abnormal or Doubtful)** |
| --- | --- | --- | --- | --- |
|  | Model 3 | Model 3 | Model 3 | Model 3 |
|  | OR [CI] | OR [CI] | OR [CI] | OR [CI] |
| Sex of Child |  |  |  |  |
| Male | 1.39^***^ [1.20,1.62] | 2.49^***^ [2.29,2.70] | 2.19^***^ [1.92,2.50] | 2.90^***^ [2.48,3.39] |
| Female | 1.00 | 1.00 | 1.00 | 1.00 |
| Birthweight Percentile |  |  |  |  |
| 0-3 | 3.06^***^ [2.15,4.36] | 1.77^***^ [1.44,2.18] | 2.26^***^ [1.63,3.13] | 3.01^***^ [2.09,4.32] |
| 4-10 | 1.84^***^ [1.42,2.39] | 1.27^**^ [1.09,1.47] | 1.54^***^ [1.23,1.94] | 1.45^**^ [1.10,1.92] |
| 11-20 | 1.36^*^ [1.05,1.75] | 1.09 [0.96,1.25] | 1.19 [0.96,1.48] | 1.43^**^ [1.12,1.82] |
| 21-80 | 1.00 | 1.00 | 1.00 | 1.00 |
| 81-90 | 0.83 [0.63,1.08] | 1.06 [0.93,1.21] | 1.06 [0.85,1.33] | 1.06 [0.82,1.36] |
| 91-97 | 0.85 [0.63,1.15] | 1.14 [0.97,1.32] | 0.97 [0.73,1.27] | 0.81 [0.59,1.12] |
| 98-100 | 0.93 [0.59,1.46] | 0.99 [0.79,1.23] | 0.93 [0.61,1.42] | 0.67 [0.40,1.11] |
| Estimated Gestation |  |  |  |  |
| Full Term (>36 weeks) | 1.00 | 1.00 | 1.00 | 1.00 |
| Preterm (32-36 weeks) | 2.70^***^ [2.01,3.63] | 1.79^***^ [1.52,2.11] | 2.28^***^ [1.75,2.95] | 2.04^***^ [1.53,2.72] |
| Maternal Smoking |  |  |  |  |
| Never | 1.00 | 1.00 | 1.00 | 1.00 |
| Current | 0.96 [0.80,1.14] | 1.25^***^ [1.14,1.37] | 1.60^***^ [1.38,1.86] | 1.51^***^ [1.27,1.80] |
| Former | 0.85 [0.65,1.13] | 0.97 [0.84,1.12] | 1.22 [0.97,1.55] | 1.08 [0.83,1.41] |
| Not known | 1.16 [0.76,1.76] | 1.25 [0.99,1.57] | 1.50^*^ [1.04,2.17] | 1.32 [0.83,2.09] |
| Model of Delivery |  |  |  |  |
| Normal | 1.00 | 1.00 | 1.00 | 1.00 |
| Other | 1.26^**^ [1.07,1.48] | 1.02 [0.94,1.11] | 1.05 [0.91,1.22] | 1.00 [0.85,1.19] |
| APGAR | 0.90^*^ [0.82,0.98] | 0.93^***^ [0.89,0.97] | 0.90^**^ [0.84,0.96] | 0.87^***^ [0.81,0.95] |
| Parity |  |  |  |  |
| Multiparous | 1.00 | 1.00 | 1.00 | 1.00 |
| Nulliparous | 0.59^***^ [0.50,0.71] | 0.53^***^ [0.49,0.58] | 0.63^***^ [0.55,0.74] | 0.64^***^ [0.54,0.76] |

*Exponentiated coefficients; 95% confidence intervals in brackets*

*^*^ p < 0.05, ^**^ p < 0.01, ^***^ p < 0.001*

Source: SLS

Model 3 also includes controls for year of admission, maternal age, maternal height, and pre-eclampsia indicator.

**Table S5:** Random effects logistic regression models of development measures with fetal environment (including birthweight).

|  | **Gross Motor Skills**  **(Abnormal or Doubtful)** | **Hearing and Language**  **(Abnormal or Doubtful)** | **Vision & Fine Motor Skills**  **(Abnormal or Doubtful)** | **Social**  **(Abnormal or Doubtful)** |
| --- | --- | --- | --- | --- |
|  | Model 3 | Model 3 | Model 3 | Model 3 |
|  | OR [CI] | OR [CI] | OR [CI] | OR [CI] |
| Sex of Child |  |  |  |  |
| Male | 1.42^***^ [1.22,1.65] | 2.50^***^ [2.31,2.72] | 2.24^***^ [1.96,2.56] | 2.92^***^ [2.50,3.42] |
| Female | 1.00 | 1.00 | 1.00 | 1.00 |
| Birthweight |  |  |  |  |
| Greater than 2500g | 1.00 | 1.00 | 1.00 | 1.00 |
| Low (1500g-2500g) | 3.36^***^ [2.42,4.68] | 1.65^***^ [1.34,2.02] | 2.53^***^ [1.85,3.45] | 2.55^***^ [1.78,3.63] |
| Very low (<1500g) | 8.56^***^ [3.53,20.78] | 2.31^**^ [1.25,4.26] | 5.30^***^ [2.01,14.01] | 6.42^***^ [2.21,18.68] |
| Estimated Gestation |  |  |  |  |
| Full Term (>36 weeks) | 1.00 | 1.00 | 1.00 | 1.00 |
| Preterm (32-36 weeks) | 1.36 [0.94,1.97] | 1.39^***^ [1.15,1.68] | 1.38 [1.00,1.92] | 1.21 [0.86,1.71] |
| Very preterm (<32 weeks) | 2.19 [0.94,5.08] | 1.39 [0.78,2.47] | 1.74 [0.68,4.48] | 1.17 [0.42,3.26] |
| Maternal Smoking |  |  |  |  |
| Never | 1.00 | 1.00 | 1.00 | 1.00 |
| Current | 1.01 [0.85,1.20] | 1.26^***^ [1.16,1.38] | 1.62^***^ [1.40,1.88] | 1.59^***^ [1.33,1.89] |
| Former | 0.86 [0.65,1.13] | 0.97 [0.84,1.12] | 1.22 [0.97,1.55] | 1.09 [0.83,1.42] |
| Not known | 1.11 [0.73,1.69] | 1.24 [0.99,1.55] | 1.50^*^ [1.04,2.16] | 1.30 [0.82,2.05] |
| Model of Delivery |  |  |  |  |
| Normal | 1.00 | 1.00 | 1.00 | 1.00 |
| Other | 1.19^*^ [1.01,1.40] | 1.01 [0.92,1.10] | 1.01 [0.87,1.16] | 0.96 [0.81,1.14] |
| APGAR | 0.92 [0.84,1.00] | 0.94^**^ [0.90,0.98] | 0.90^**^ [0.84,0.97] | 0.89^**^ [0.82,0.96] |
| Parity |  |  |  |  |
| Multiparous | 1.00 | 1.00 | 1.00 | 1.00 |
| Nulliparous | 0.64^***^ [0.54,0.75] | 0.54^***^ [0.49,0.59] | 0.66^***^ [0.57,0.77] | 0.69^***^ [0.58,0.81] |

*Exponentiated coefficients; 95% confidence intervals in brackets*

*^*^ p < 0.05, ^**^ p < 0.01, ^***^ p < 0.001*

Source: SLS

Model 3 also includes controls for year of admission, maternal age, maternal height, and pre-eclampsia indicator.

**Table S6:** Random effects logistic regression models of development measures with socioeconomic position and fetal environment (including estimates not reported in table 2).

|  | **Gross Motor Skills**  **(Abnormal or Doubtful)** | | **Hearing and Language**  **(Abnormal or Doubtful)** | | **Vision & Fine Motor Skills**  **(Abnormal or Doubtful)** | | **Social**  **(Abnormal or Doubtful)** | |
| --- | --- | --- | --- | --- | --- | --- | --- | --- |
|  | Model 1 | Model 2^a^ | Model 1 | Model 2^a^ | Model 1 | Model 2^a^ | Model 1 | Model 2^a^ |
|  | OR [CI] | OR [CI] | OR [CI] | OR [CI] | OR [CI] | OR [CI] | OR [CI] | OR [CI] |
| Marital Status |  |  |  |  |  |  |  |  |
| Married | 1.00 | 1.00 | 1.00 | 1.00 | 1.00 | 1.00 | 1.00 | 1.00 |
| Not married | 1.10 [0.94,1.30] | 1.09 [0.92,1.30] | 0.93 [0.85,1.01] | 1.07 [0.97,1.17] | 1.16^*^ [1.01,1.34] | 1.28^**^ [1.10,1.49] | 1.09 [0.92,1.29] | 1.18 [0.98,1.41] |
| Sex of Child |  |  |  |  |  |  |  |  |
| Male |  | 1.39^***^ [1.20,1.62] |  | 2.50^***^ [2.30,2.71] |  | 2.19^***^ [1.92,2.50] |  | 2.89^***^ [2.47,3.38] |
| Female |  | 1.00 |  | 1.00 |  | 1.00 |  | 1.00 |
| Maternal Smoking |  |  |  |  |  |  |  |  |
| Never |  | 1.00 |  | 1.00 |  | 1.00 |  | 1.00 |
| Current |  | 0.81^*^ [0.67,0.97] |  | 1.04 [0.94,1.14] |  | 1.18^*^ [1.01,1.38] |  | 1.15 [0.96,1.39] |
| Former |  | 0.81 [0.61,1.06] |  | 0.90 [0.78,1.05] |  | 1.11 [0.87,1.40] |  | 1.00 [0.76,1.30] |
| Not known |  | 1.09 [0.71,1.65] |  | 1.19 [0.95,1.49] |  | 1.35 [0.93,1.94] |  | 1.21 [0.76,1.92] |
| Model of Delivery |  |  |  |  |  |  |  |  |
| Normal |  | 1.00 |  | 1.00 |  | 1.00 |  | 1.00 |
| Other |  | 1.26^**^ [1.07,1.49] |  | 1.02 [0.94,1.11] |  | 1.07 [0.92,1.23] |  | 1.01 [0.86,1.20] |
| APGAR |  | 0.90^*^ [0.83,0.98] |  | 0.93^**^ [0.90,0.97] |  | 0.90^**^ [0.85,0.97] |  | 0.88^**^ [0.82,0.95] |
| Parity |  |  |  |  |  |  |  |  |
| Multiparous |  | 1.00 |  | 1.00 |  | 1.00 |  | 1.00 |
| Nulliparous |  | 0.64^***^ [0.54,0.76] |  | 0.58^***^ [0.53,0.64] |  | 0.73^***^ [0.63,0.85] |  | 0.74^***^ [0.62,0.88] |
| *Observations (n)* | 114398 | 114398 | 114398 | 114398 | 93036 | 93036 | 93036 | 93036 |
| *Children (n groups)* | 32076 | 32076 | 32076 | 32076 | 31572 | 31572 | 31572 | 31572 |
| *Parents (n clusters)* | 27313 | 27313 | 27313 | 27313 | 27010 | 27010 | 27010 | 27010 |
| *Mean number of reviews* | 3.57 | 3.57 | 3.57 | 3.57 | 2.95 | 2.95 | 2.95 | 2.95 |
| *SD of residuals within groups* | 2.18 | 2.10 | 1.48 | 1.37 | 1.91 | 1.85 | 2.14 | 2.04 |
| *Intraclass Correlation* | 0.59 | 0.57 | 0.40 | 0.36 | 0.53 | 0.51 | 0.58 | 0.56 |
| *AIC* | 14466.08 | 14320.44 | 37300.52 | 36433.90 | 15998.60 | 15740.23 | 13174.78 | 12900.50 |
| *BIC* | 14620.44 | 14822.10 | 37454.87 | 36935.57 | 16149.66 | 16231.15 | 13325.83 | 13391.42 |
| *Log Likelihood* | -7217.04 | -7108.22 | -18634.26 | -18164.95 | -7983.30 | -7818.12 | -6571.39 | -6398.25 |
| *Model Degrees of Freedom* | 14 | 50 | 14 | 50 | 14 | 50 | 14 | 50 |

*Exponentiated coefficients; 95% confidence intervals in brackets*

*^*^ p < 0.05, ^**^ p < 0.01, ^***^ p < 0.001*

Source: SLS

a Model 1 also includes parent NS-SEC, and parent highest qualification.

b Model 2 also includes parent NS-SEC, and parent highest qualification, birthweight percentile, year of admission, maternal age, maternal height, and pre-eclampsia indicator.

**Table S7:** Random effects logistic regression models of development measures with socioeconomic position abd fetal environment (sensitivity analysis using birthweight).

|  | **Gross Motor Skills**  **(Abnormal or Doubtful)** | | **Hearing and Language**  **(Abnormal or Doubtful)** | | **Vision & Fine Motor Skills**  **(Abnormal or Doubtful)** | | **Social**  **(Abnormal or Doubtful)** | |
| --- | --- | --- | --- | --- | --- | --- | --- | --- |
|  | Model 1 | Model 2^a^ | Model 1 | Model 2^a^ | Model 1 | Model 2^a^ | Model 1 | Model 2^a^ |
|  | OR [CI] | OR [CI] | OR [CI] | OR [CI] | OR [CI] | OR [CI] | OR [CI] | OR [CI] |
| Parent NS-SEC |  |  |  |  |  |  |  |  |
| 1.1 | 1.00 | 1.00 | 1.00 | 1.00 | 1.00 | 1.00 | 1.00 | 1.00 |
| 1.2 | 1.14 [0.74,1.76] | 1.13 [0.74,1.75] | 1.02 [0.80,1.30] | 1.05 [0.83,1.34] | 1.38 [0.89,2.14] | 1.46 [0.94,2.26] | 1.44 [0.86,2.40] | 1.50 [0.90,2.51] |
| 2 | 1.16 [0.80,1.67] | 1.17 [0.82,1.69] | 1.23^*^ [1.01,1.50] | 1.24^*^ [1.02,1.50] | 1.56^*^ [1.09,2.23] | 1.57^*^ [1.10,2.25] | 1.80^**^ [1.18,2.75] | 1.80^**^ [1.18,2.75] |
| 3 | 1.06 [0.71,1.58] | 1.10 [0.73,1.63] | 1.21 [0.98,1.51] | 1.25^*^ [1.01,1.55] | 1.46 [0.99,2.14] | 1.49^*^ [1.01,2.18] | 1.53 [0.97,2.43] | 1.60^*^ [1.01,2.54] |
| 4 | 1.00 [0.63,1.60] | 1.00 [0.63,1.58] | 1.52^**^ [1.19,1.96] | 1.44^**^ [1.13,1.85] | 1.69^*^ [1.09,2.61] | 1.64^*^ [1.07,2.53] | 2.44^***^ [1.47,4.04] | 2.39^***^ [1.45,3.95] |
| 5 | 1.07 [0.70,1.65] | 1.05 [0.68,1.63] | 1.54^***^ [1.23,1.94] | 1.47^***^ [1.17,1.84] | 1.62^*^ [1.07,2.44] | 1.53^*^ [1.01,2.32] | 1.99^**^ [1.22,3.24] | 1.95^**^ [1.20,3.18] |
| 6 | 1.22 [0.81,1.84] | 1.28 [0.85,1.93] | 1.69^***^ [1.36,2.10] | 1.60^***^ [1.29,1.99] | 2.26^***^ [1.54,3.31] | 2.21^***^ [1.51,3.24] | 2.55^***^ [1.62,4.02] | 2.56^***^ [1.62,4.04] |
| 7 | 1.44 [0.93,2.23] | 1.50 [0.96,2.33] | 1.66^***^ [1.31,2.10] | 1.56^***^ [1.23,1.97] | 2.44^***^ [1.62,3.67] | 2.37^***^ [1.57,3.56] | 2.63^***^ [1.62,4.29] | 2.57^***^ [1.58,4.19] |
| Never worked and LTU | 1.79^*^ [1.09,2.92] | 1.76^*^ [1.07,2.91] | 2.14^***^ [1.64,2.80] | 1.91^***^ [1.46,2.51] | 3.79^***^ [2.44,5.88] | 3.46^***^ [2.21,5.42] | 4.41^***^ [2.63,7.38] | 4.11^***^ [2.43,6.95] |
| FT Students | 1.54 [0.80,2.98] | 1.67 [0.87,3.21] | 1.48^*^ [1.03,2.13] | 1.77^**^ [1.23,2.54] | 1.84 [1.00,3.39] | 2.13^*^ [1.15,3.94] | 2.02 [0.97,4.21] | 2.39^*^ [1.14,4.99] |
| Parental Highest Qualification |  |  |  |  |  |  |  |  |
| Degree | 1.00 | 1.00 | 1.00 | 1.00 | 1.00 | 1.00 | 1.00 | 1.00 |
| HNC/HND | 1.04 [0.79,1.38] | 1.06 [0.80,1.41] | 1.08 [0.93,1.25] | 1.05 [0.91,1.22] | 1.17 [0.90,1.52] | 1.13 [0.87,1.47] | 1.02 [0.76,1.36] | 1.01 [0.76,1.35] |
| Highers/CSYS | 1.06 [0.82,1.36] | 1.07 [0.84,1.38] | 1.13 [0.98,1.29] | 1.11 [0.97,1.27] | 1.24 [0.98,1.57] | 1.19 [0.94,1.50] | 0.91 [0.70,1.19] | 0.91 [0.70,1.19] |
| O Grade/S Grade | 1.33^*^ [1.05,1.70] | 1.35^*^ [1.06,1.73] | 1.40^***^ [1.23,1.60] | 1.29^***^ [1.13,1.47] | 1.55^***^ [1.24,1.92] | 1.38^**^ [1.11,1.72] | 1.14 [0.89,1.47] | 1.07 [0.83,1.37] |
| No Qualifications | 1.61^**^ [1.21,2.15] | 1.62^**^ [1.21,2.18] | 1.82^***^ [1.55,2.13] | 1.56^***^ [1.33,1.83] | 2.36^***^ [1.83,3.04] | 1.95^***^ [1.51,2.51] | 2.04^***^ [1.53,2.72] | 1.77^***^ [1.32,2.37] |
| Marital Status |  |  |  |  |  |  |  |  |
| Married | 1.00 | 1.00 | 1.00 | 1.00 | 1.00 | 1.00 | 1.00 | 1.00 |
| Not married | 1.09 [0.93,1.28] | 1.08 [0.91,1.29] | 0.93 [0.86,1.02] | 1.07 [0.97,1.17] | 1.15^*^ [1.00,1.33] | 1.27^**^ [1.09,1.48] | 1.08 [0.91,1.27] | 1.17 [0.98,1.40] |
| Sex of Child |  |  |  |  |  |  |  |  |
| Male |  | 1.42^***^ [1.23,1.65] |  | 2.51^***^ [2.31,2.72] |  | 2.23^***^ [1.95,2.55] |  | 2.91^***^ [2.49,3.40] |
| Female |  | 1.00 |  | 1.00 |  | 1.00 |  | 1.00 |
| Birthweight |  |  |  |  |  |  |  |  |
| Greater than 2500g |  | 1.00 |  | 1.00 |  | 1.00 |  | 1.00 |
| Low (1500g-2500g) |  | 3.24^***^ [2.33,4.50] |  | 1.59^***^ [1.29,1.95] |  | 2.35^***^ [1.73,3.20] |  | 2.40^***^ [1.68,3.43] |
| Very low (<1500g) |  | 8.56^***^ [3.60,20.38] |  | 2.40^**^ [1.31,4.38] |  | 5.36^***^ [2.09,13.75] |  | 6.75^***^ [2.44,18.71] |
| Estimated Gestation |  |  |  |  |  |  |  |  |
| Full Term (>36 weeks) |  | 1.00 |  | 1.00 |  | 1.00 |  | 1.00 |
| Preterm (32-36 weeks) |  | 1.36 [0.94,1.96] |  | 1.38^***^ [1.14,1.67] |  | 1.39^*^ [1.00,1.92] |  | 1.20 [0.85,1.70] |
| Very preterm (<32 weeks) |  | 2.15 [0.94,4.88] |  | 1.32 [0.75,2.32] |  | 1.68 [0.67,4.18] |  | 1.08 [0.41,2.85] |
| Maternal Smoking |  |  |  |  |  |  |  |  |
| Never |  | 1.00 |  | 1.00 |  | 1.00 |  | 1.00 |
| Current |  | 0.84 [0.70,1.00] |  | 1.04 [0.95,1.14] |  | 1.18^*^ [1.01,1.37] |  | 1.19 [0.99,1.42] |
| Former |  | 0.81 [0.62,1.07] |  | 0.91 [0.78,1.05] |  | 1.10 [0.87,1.39] |  | 1.00 [0.77,1.30] |
| Not known |  | 1.05 [0.69,1.59] |  | 1.18 [0.94,1.48] |  | 1.36 [0.95,1.95] |  | 1.19 [0.75,1.88] |
| Model of Delivery |  |  |  |  |  |  |  |  |
| Normal |  | 1.00 |  | 1.00 |  | 1.00 |  | 1.00 |
| Other |  | 1.19^*^ [1.02,1.40] |  | 1.01 [0.93,1.10] |  | 1.02 [0.88,1.18] |  | 0.98 [0.83,1.16] |
| APGAR |  | 0.92 [0.85,1.00] |  | 0.94^**^ [0.91,0.98] |  | 0.91^**^ [0.85,0.97] |  | 0.90^**^ [0.83,0.97] |
| Parity |  |  |  |  |  |  |  |  |
| Multiparous |  | 1.00 |  | 1.00 |  | 1.00 |  | 1.00 |
| Nulliparous |  | 0.69^***^ [0.58,0.82] |  | 0.59^***^ [0.54,0.65] |  | 0.76^***^ [0.66,0.88] |  | 0.79^**^ [0.66,0.93] |
| *Observations [n)* | 114928 | 114928 | 114928 | 114928 | 93481 | 93481 | 93481 | 93481 |
| *Children [n groups)* | 32238 | 32238 | 32238 | 32238 | 31731 | 31731 | 31731 | 31731 |
| *Parents [n clusters)* | 27438 | 27438 | 27438 | 27438 | 27135 | 27135 | 27135 | 27135 |
| *Mean number of reviews* | 3.56 | 3.56 | 3.56 | 3.56 | 2.95 | 2.95 | 2.95 | 2.95 |
| *SD of residuals within groups* | 2.19 | 2.09 | 1.48 | 1.37 | 1.93 | 1.86 | 2.16 | 2.06 |
| *Intraclass Correlation* | 0.59 | 0.57 | 0.40 | 0.36 | 0.53 | 0.51 | 0.59 | 0.56 |
| *AIC* | 14882.91 | 14654.05 | 37665.23 | 36780.55 | 16311.17 | 15989.04 | 13383.47 | 13099.16 |
| *BIC* | 15037.35 | 15127.00 | 37819.66 | 37253.50 | 16462.30 | 16451.87 | 13534.59 | 13561.99 |
| *Log Likelihood* | -7425.46 | -7278.02 | -18816.61 | -18341.28 | -8139.58 | -7945.52 | -6675.73 | -6500.58 |
| *Model Degrees of Freedom* | 14 | 47 | 14 | 47 | 14 | 47 | 14 | 47 |

Exponentiated coefficients; 95% confidence intervals in brackets

^*^ *p* < 0.05, ^**^ *p* < 0.01, ^***^ *p* < 0.001

Source: SLS

a Model 2 also includes controls for year of admission, maternal age, maternal height, and pre-eclampsia indicator

**Table S8:** Random effects logistic regression models of development measures with socioeconomic position and fetal environment for males and females (1).

|  | **Male** | | **Female** | | **Male** | | **Female** | |
| --- | --- | --- | --- | --- | --- | --- | --- | --- |
|  | **Gross Motor Skills**  **(Abnormal or Doubtful)** | | **Gross Motor Skills**  **(Abnormal or Doubtful)** | | **Hearing and Language**  **(Abnormal or Doubtful)** | | **Hearing and Language**  **(Abnormal or Doubtful)** | |
|  | Model 1 | Model 2^a^ | Model 1 | Model 2^a^ | Model 1 | Model 2^a^ | Model 1 | Model 2^a^ |
|  | OR [CI] | OR [CI] | OR [CI] | OR [CI] | OR [CI] | OR [CI] | OR [CI] | OR [CI] |
| Parent NS-SEC |  |  |  |  |  |  |  |  |
| 1.1 | 1.00 | 1.00 | 1.00 | 1.00 | 1.00 | 1.00 | 1.00 | 1.00 |
| 1.2 | 1.34 [0.77,2.32] | 1.25 [0.72,2.16] | 0.99 [0.53,1.88] | 0.95 [0.51,1.80] | 0.95 [0.71,1.26] | 0.95 [0.71,1.25] | 1.30 [0.83,2.04] | 1.30 [0.83,2.03] |
| 2 | 1.31 [0.83,2.08] | 1.32 [0.83,2.08] | 0.97 [0.58,1.64] | 0.96 [0.57,1.62] | 1.24 [1.00,1.54] | 1.24 [1.00,1.54] | 1.26 [0.87,1.84] | 1.26 [0.87,1.84] |
| 3 | 1.12 [0.67,1.87] | 1.11 [0.67,1.85] | 0.99 [0.56,1.76] | 1.04 [0.58,1.84] | 1.09 [0.86,1.40] | 1.10 [0.86,1.40] | 1.61* [1.07,2.41] | 1.63* [1.08,2.44] |
| 4 | 1.26 [0.69,2.29] | 1.25 [0.69,2.28] | 0.67 [0.32,1.41] | 0.64 [0.30,1.34] | 1.33 [1.00,1.78] | 1.25 [0.94,1.67] | 2.03** [1.28,3.21] | 1.89** [1.19,3.00] |
| 5 | 1.23 [0.70,2.16] | 1.18 [0.67,2.08] | 0.81 [0.42,1.56] | 0.79 [0.41,1.53] | 1.63*** [1.26,2.11] | 1.51** [1.16,1.96] | 1.46 [0.94,2.27] | 1.38 [0.89,2.15] |
| 6 | 1.29 [0.76,2.18] | 1.28 [0.76,2.16] | 1.18 [0.66,2.13] | 1.18 [0.65,2.14] | 1.72*** [1.35,2.20] | 1.59*** [1.25,2.03] | 1.71* [1.13,2.59] | 1.62* [1.06,2.47] |
| 7 | 1.89* [1.09,3.30] | 1.86* [1.07,3.24] | 1.01 [0.53,1.92] | 1.04 [0.54,1.99] | 1.68*** [1.28,2.19] | 1.52** [1.16,1.99] | 1.74* [1.11,2.70] | 1.65* [1.05,2.59] |
| Never worked and LTU | 1.89 [1.00,3.57] | 1.84 [0.96,3.52] | 1.47 [0.70,3.06] | 1.38 [0.65,2.92] | 1.83*** [1.34,2.52] | 1.57** [1.14,2.17] | 2.94*** [1.83,4.75] | 2.73*** [1.67,4.47] |
| FT Students | 1.48 [0.61,3.56] | 1.60 [0.66,3.87] | 1.72 [0.69,4.31] | 1.68 [0.66,4.30] | 1.55* [1.00,2.39] | 1.78* [1.14,2.76] | 1.59 [0.85,2.98] | 1.88 [0.99,3.54] |
| Parental Highest Qualification |  |  |  |  |  |  |  |  |
| Degree | 1.00 | 1.00 | 1.00 | 1.00 | 1.00 | 1.00 | 1.00 | 1.00 |
| HNC/HND | 1.04 [0.73,1.49] | 1.03 [0.72,1.47] | 1.06 [0.69,1.62] | 1.05 [0.69,1.61] | 1.14 [0.96,1.36] | 1.11 [0.93,1.32] | 0.92 [0.70,1.21] | 0.92 [0.70,1.21] |
| Highers/CSYS | 1.13 [0.82,1.57] | 1.17 [0.85,1.62] | 1.01 [0.68,1.48] | 0.99 [0.67,1.47] | 1.20* [1.02,1.41] | 1.16 [0.99,1.37] | 1.02 [0.80,1.31] | 1.00 [0.78,1.28] |
| O Grade/S Grade | 1.36 [1.00,1.86] | 1.40* [1.02,1.92] | 1.31 [0.91,1.89] | 1.26 [0.87,1.83] | 1.40*** [1.20,1.64] | 1.27** [1.09,1.49] | 1.40** [1.11,1.78] | 1.30* [1.03,1.65] |
| No Qualifications | 1.50* [1.02,2.22] | 1.50* [1.01,2.22] | 1.81* [1.15,2.84] | 1.72* [1.08,2.72] | 1.87*** [1.56,2.26] | 1.58*** [1.31,1.91] | 1.78*** [1.34,2.36] | 1.49** [1.12,1.99] |
| Birthweight Percentile |  |  |  |  |  |  |  |  |
| 0-3 |  | 2.51*** [1.57,4.01] |  | 3.43*** [2.09,5.65] |  | 1.56*** [1.22,1.99] |  | 1.92*** [1.33,2.78] |
| 4-10 |  | 2.08*** [1.49,2.90] |  | 1.40 [0.92,2.15] |  | 1.23* [1.03,1.46] |  | 1.20 [0.92,1.57] |
| 11-20 |  | 1.23 [0.90,1.70] |  | 1.44* [1.01,2.07] |  | 1.03 [0.88,1.20] |  | 1.13 [0.89,1.43] |
| 21-80 |  | 1.00 |  | 1.00 |  | 1.00 |  | 1.00 |
| 81-90 |  | 1.04 [0.75,1.43] |  | 0.58* [0.37,0.91] |  | 1.06 [0.91,1.24] |  | 1.08 [0.84,1.38] |
| 91-97 |  | 0.82 [0.55,1.22] |  | 0.87 [0.55,1.38] |  | 1.12 [0.93,1.34] |  | 1.22 [0.93,1.59] |
| 98-100 |  | 1.01 [0.58,1.78] |  | 0.83 [0.42,1.65] |  | 1.04 [0.80,1.36] |  | 0.93 [0.63,1.39] |
| Estimated Gestation |  |  |  |  |  |  |  |  |
| Full Term (>36 weeks) |  | 1.00 |  | 1.00 |  | 1.00 |  | 1.00 |
| Preterm (32-36 weeks) |  | 2.69*** [1.88,3.84] |  | 2.71*** [1.72,4.29] |  | 1.53*** [1.27,1.85] |  | 2.27*** [1.71,3.01] |

*Exponentiated coefficients; 95% confidence intervals in brackets*

*^*^ p < 0.05, ^**^ p < 0.01, ^***^ p < 0.001*

Source: SLS

a Model 1 also includes maternal marital status

b Model 2 also includes controls for maternal marital status, sex, maternal smoking history, mode of delivery, APGAR score, parity, year of admission, maternal age, maternal height, and pre-eclampsia indicator.

It was necessary to relax the assumption of familial clustering for the models estimating gross motor skills to ensure the model converged.

**Table S9:** Random effects logistic regression models of development measures with socioeconomic position and fetal environment for males and females (2).

|  | **Male** | | **Female** | | **Male** | | **Female** | |
| --- | --- | --- | --- | --- | --- | --- | --- | --- |
|  | **Vision & Fine Motor Skills**  **(Abnormal or Doubtful)** | | **Vision & Fine Motor Skills**  **(Abnormal or Doubtful)** | | **Social**  **(Abnormal or Doubtful)** | | **Social**  **(Abnormal or Doubtful)** | |
|  | Model 1 | Model 2^a^ | Model 1 | Model 2^a^ | Model 1 | Model 2^a^ | Model 1 | Model 2^a^ |
|  | OR [CI] | OR [CI] | OR [CI] | OR [CI] | OR [CI] | OR [CI] | OR [CI] | OR [CI] |
| Parent NS-SEC |  |  |  |  |  |  |  |  |
| 1.1 | 1.00 | 1.00 | 1.00 | 1.00 | 1.00 | 1.00 | 1.00 | 1.00 |
| 1.2 | 1.32 [0.79,2.18] | 1.34 [0.81,2.22] | 1.59 [0.74,3.40] | 1.65 [0.77,3.53] | 1.25 [0.70,2.22] | 1.22 [0.69,2.15] | 2.33 [0.80,6.78] | 2.39 [0.82,6.92] |
| 2 | 1.39 [0.92,2.11] | 1.41 [0.93,2.14] | 1.78 [0.93,3.40] | 1.77 [0.93,3.39] | 1.63* [1.02,2.59] | 1.62* [1.02,2.57] | 2.21 [0.87,5.62] | 2.18 [0.86,5.51] |
| 3 | 1.25 [0.79,1.96] | 1.23 [0.78,1.93] | 1.89 [0.95,3.79] | 1.95 [0.97,3.90] | 1.21 [0.72,2.01] | 1.2 [0.73,2.00] | 2.76* [1.04,7.37] | 2.90* [1.09,7.72] |
| 4 | 1.49 [0.89,2.50] | 1.45 [0.86,2.42] | 1.95 [0.88,4.30] | 1.92 [0.87,4.24] | 2.01* [1.15,3.51] | 1.97* [1.13,3.43] | 3.65* [1.27,10.50] | 3.47* [1.21,9.97] |
| 5 | 1.68* [1.03,2.72] | 1.56 [0.96,2.54] | 1.37 [0.63,2.95] | 1.28 [0.59,2.77] | 1.92* [1.12,3.29] | 1.86* [1.08,3.17] | 2.3 [0.80,6.56] | 2.15 [0.75,6.20] |
| 6 | 1.98** [1.26,3.11] | 1.87** [1.19,2.95] | 2.76** [1.38,5.55] | 2.61** [1.29,5.26] | 2.17** [1.32,3.58] | 2.11** [1.28,3.46] | 3.77** [1.40,10.12] | 3.43* [1.27,9.29] |
| 7 | 2.34*** [1.45,3.78] | 2.21** [1.37,3.58] | 2.47* [1.18,5.17] | 2.36* [1.12,4.96] | 2.60*** [1.52,4.44] | 2.49*** [1.46,4.24] | 2.6 [0.91,7.45] | 2.45 [0.84,7.13] |
| Never worked and LTU | 3.24*** [1.92,5.46] | 2.93*** [1.72,4.98] | 4.42*** [2.01,9.73] | 4.00*** [1.79,8.94] | 3.79*** [2.15,6.69] | 3.51*** [1.98,6.23] | 5.38** [1.80,16.07] | 4.94** [1.62,15.09] |
| FT Students | 1.89 [0.90,3.98] | 2.07 [0.97,4.40] | 1.87 [0.60,5.86] | 2.19 [0.68,7.03] | 1.56 [0.64,3.82] | 1.75 [0.71,4.27] | 4.15* [1.07,16.07] | 4.63* [1.20,17.83] |
| Parental Highest Qualification |  |  |  |  |  |  |  |  |
| Degree | 1.00 | 1.00 | 1.00 | 1.00 | 1.00 | 1.00 | 1.00 | 1.00 |
| HNC/HND | 1.26 [0.93,1.72] | 1.21 [0.89,1.65] | 1.02 [0.65,1.60] | 0.97 [0.62,1.52] | 1.09 [0.78,1.52] | 1.08 [0.78,1.51] | 0.88 [0.50,1.52] | 0.84 [0.48,1.45] |
| Highers/CSYS | 1.40* [1.06,1.86] | 1.37* [1.04,1.82] | 0.99 [0.66,1.49] | 0.91 [0.60,1.37] | 0.93 [0.68,1.26] | 0.93 [0.68,1.26] | 0.98 [0.59,1.65] | 0.92 [0.55,1.53] |
| O Grade/S Grade | 1.66*** [1.27,2.18] | 1.51** [1.15,1.98] | 1.36 [0.93,1.99] | 1.17 [0.80,1.72] | 1.25 [0.94,1.66] | 1.20 [0.90,1.60] | 0.98 [0.60,1.61] | 0.83 [0.51,1.36] |
| No Qualifications | 2.44*** [1.78,3.34] | 2.05*** [1.49,2.82] | 2.41*** [1.55,3.74] | 1.80* [1.15,2.83] | 2.07*** [1.49,2.88] | 1.83*** [1.31,2.57] | 2.24** [1.28,3.93] | 1.71 [0.98,2.98] |
| Birthweight Percentile |  |  |  |  |  |  |  |  |
| 0-3 |  | 2.06*** [1.40,3.02] |  | 2.11** [1.26,3.52] |  | 2.01** [1.28,3.16] |  | 4.78*** [2.60,8.78] |
| 4-10 |  | 1.50** [1.14,1.99] |  | 1.31 [0.87,1.98] |  | 1.30 [0.94,1.80] |  | 1.45 [0.87,2.42] |
| 11-20 |  | 0.99 [0.76,1.29] |  | 1.42 [1.00,2.02] |  | 1.25 [0.94,1.65] |  | 1.72* [1.12,2.62] |
| 21-80 |  | 1.00 |  | 1.00 |  | 1.00 |  | 1.00 |
| 81-90 |  | 1.14 [0.87,1.48] |  | 0.98 [0.66,1.47] |  | 1.10 [0.83,1.45] |  | 1.03 [0.62,1.73] |
| 91-97 |  | 0.91 [0.66,1.27] |  | 1.13 [0.71,1.80] |  | 0.84 [0.59,1.21] |  | 0.72 [0.38,1.36] |
| 98-100 |  | 0.89 [0.53,1.50] |  | 1.10 [0.55,2.21] |  | 0.88 [0.50,1.53] |  | 0.35 [0.11,1.15] |
| Estimated Gestation |  |  |  |  |  |  |  |  |
| Full Term (>36 weeks) |  | 1.00 |  | 1.00 |  | 1.00 |  | 1.00 |
| Preterm (32-36 weeks) |  | 1.95*** [1.43,2.66] |  | 2.82*** [1.81,4.37] |  | 1.66** [1.18,2.33] |  | 3.03*** [1.79,5.13] |

*Exponentiated coefficients; 95% confidence intervals in brackets*

*^*^ p < 0.05, ^**^ p < 0.01, ^***^ p < 0.001*

Source: SLS

a Model 1 also includes maternal marital status

b Model 2 also includes controls for maternal marital status, sex, maternal smoking history, mode of delivery, APGAR score, parity, year of admission, maternal age, maternal height, and pre-eclampsia indicator.

It was necessary to relax the assumption of familial clustering for the models estimating vision and fine motor skills to ensure the model converged.

We also tested for the following interactions effects for each of the child development indicators using Bayesian Information Criterion (BIC):

- Parental NS-SEC and birthweight
- Parental NS-SEC and birthweight percentile
- Parental NS-SEC and estimated gestational age
- Parental highest qualification and birthweight
- Parental highest qualification and birthweight percentile
- Parental highest qualification and estimated gestational age

These have not been reported because the interaction models were not informative and did not represent an improvement on the main effects model in each case.
